# First Report of Olive Branch Dieback in Croatia Caused by *Cytospora pruinosa* Défago

**DOI:** 10.3390/microorganisms11071679

**Published:** 2023-06-28

**Authors:** Elena Petrović, Karolina Vrandečić, Dario Ivić, Jasenka Ćosić, Sara Godena

**Affiliations:** 1 Institute of Agriculture and Tourism, Karla Huguesa 8, 52440 Poreč, Croatia; elena@iptpo.hr; 2 Faculty of Agrobiotechnical Sciences Osijek, Josip Juraj Strossmayer University of Osijek, Vladimira Preloga 1, 31000 Osijek, Croatia; kvrandecic@fazos.hr (K.V.); jcosic@fazos.hr (J.Ć.); 3 Centre for Plant Protection, Croatian Agency for Agriculture and Food, Gorice 68b, 10000 Zagreb, Croatia

**Keywords:** canker, *Cytospora* sp., fungal disease, *Olea europaea* L.

## Abstract

Olive (*Olea europaea* L.) is a very important crop grown in the Mediterranean part of Croatia. Olive branch and fruit dieback symptoms were observed in two olive orchards in Istria, Croatia. The samples from symptomatic trees were collected and brought to the laboratory for analysis. Based on their morphological characterization, isolated fungi were identified as *Cytospora* sp. Two representative isolates (one per orchard) were taken for molecular analysis, and based on DNA sequence data of the ITS and TUB gene regions, and phylogenetic analysis of the sequences, the isolates were identified as *Cytospora pruinosa* Défago. To determine pathogenicity, pathogenicity tests were conducted on detached olive branches and two-year-old olive trees in the greenhouse. This is the first report of *C. pruinosa* causing olive branch and fruit dieback in Croatia.

## 1. Introduction

The olive (*Olea europaea* L.) is a medium-sized evergreen tree from the family *Oleaceae*, which integrates a unique set of morphological and developmental characteristics suited to the conditions of its Mediterranean origin [1]. The Mediterranean climate is characterized by an amount of rainfall ranging from 150 to 800 mm per year, and by the uneven distribution of rains, concentrated above all in winter and spring months [2,3]. The olive tree is very adapted to extreme environmental conditions, such as drought and high temperatures, and it is resistant to decay [2,4].

During the last decade, plantings and production of European olive (*Olea europaea* L.) have increased globally by about 10 and 20% [5]. According to the latest statistical data, worldwide production of olives is approximately 23 million tons, and it is cultivated on approximately 10 million ha [6]. In Croatia, olives are cultivated on almost 20 thousand ha with a production of 23 thousand tons of olives [6]. Olive is, along with vines, the most common crop grown in the Mediterranean part of Croatia [7]. In Istria, olive trees are known for about 2500 years [8]. Hundreds of named cultivars of both types of olives, table and oil, are grown. The most important domesticated and introduced olive cultivars in Istria (Croatia) are ‘Bjelica’, ‘Buža’, and ‘Leccino’.

Olives are susceptible to different bacterial, viral, and fungal pathogens, which can cause severe diseases of the drupe, leaves, wood, and roots [9]. Trunk pathogens can infect olive trees through wounds, and cause dieback of twigs and branches, which can lead to a reduced fruit-bearing capacity and lifespan of olive trees [9,10]. Consequently, fungal trunk diseases can cause substantial economic losses [5]. Branches affected with cankers can show symptoms such as fruit rot or twig dieback. A number of different pathogens are reported as the causal agents associated with olive cankers and twig dieback [10]. One of the most harmful olive pathogens associated with the fungal canker of olives is species from the *Botryosphaeriaceae* family. The *Botryophaeriaceae* were found to be the most prevalent fungal family causing olive twig and branch dieback in California, Italy, and Spain [9,10,11]. Olive diseases are poorly studied in Croatia, with most research focusing on practical aspects of leaf spot, caused by *Venturia oleaginea* (Castagne) Rossman & Crous, and olive knot, caused by *Pseudomonas savastanoi* (Janse 1982) Gardan et al., management [12]. Thus far, the species *Botryosphaeria dothidea* (Moug. ex Fr.) Ces. & De Not., *Diaporthe neotheicola* (Sacc.) Udayanga & Castl., *Diplodia seriata* De Notaris, *Neofusicuccoum parvum* (Pennycook & Samuels) Crous, Slippers & A.J.L. Phillips, *Phaeoacremonium iranianum* L. Mostert, Gräfenhan, W. Gams & Crous, *Phoma incompta* Sacc. & Martelli (*Comoclathris incompta* (Sacc. & Martelli) Ariyaw. & K.D. Hyde), *Pleurostomophora richardsiae* (Nannf.) Reblova & Jaklitsch, and *Verticillium dahliae* Klebahn have been described as fungal pathogens of olive in Croatia [12,13,14,15,16,17].

*Cytospora* spp. (anamorphs of *Valsa* spp.) are common inhabitants of woody plants and they include important stem and branch canker pathogens [18]. Since many different pathogens could be associated with the same syndrome, the correct diagnosis of the diseases is difficult. Diseases associated with *Cytospora* spp. have been referred to as Cytospora-, Leucostoma-, Perennial-, or Valsa canker [5,18,19,20]. There have been several reports of *Cytospora* spp. on olive trees, but there is a lack of knowledge about the diversity and biology of *Cytospora* spp. affecting olive [5]. *Cytospora* genus belongs to the order *Diaporthales*, and was introduced in 1818 with four species, namely: *C. betulina*, *C. epimyces*, *C. resinae*, and *C. ribis* [21]. The genus *Cytospora* has asexual morphs in *Valsa*, *Leucostoma*, *Valsella*, and *Valseutypella* [19]. Recently, all sexual genera were synonymized with *Valsa* as a subgenus or species [19]. This order is well known to contain endophytes, phytopathogens, and saprobes, with worldwide distribution [22,23,24]. Currently, there are 3373 described species of *Diaporthales*, in 238 genera and 11 families [25]. Many different names and name changes within *Cytospora* species have caused confusion for plant pathologists. The Index Fungorum lists 672 species of *Cytospora* and 886 species of *Valsa*. Most species are believed to be synonyms [18]. Currently, there are 173 records of *Cytospora* species in GenBank.

In the past, species identification in *Cytospora* was largely based on host affiliation, morphological characters of pycnidia/perithecia, and spore dimensions, with morphological description [26,27,28]. This morphological species approach is confounded by many morphological characters’ overlap among species and by the morphological plasticity of pycnidial locules which are affected by the host bark and cambium characteristics (e.g., stromatal arrangement in the host tissues, locular arrangement within pycnidia, locule division into chambers, independent or shared locular walls) [18,19,29,30,31]. The first molecular phylogeny of *Cytospora* was inferred from ITS sequences [29], and identifications were performed mostly based on the ITS gene region [19,32,33]. Recently, more *Cytospora* species were recognized when using analysis including multiple protein-coding loci, such as α-actin (ACT), beta-tubulin (TUB2), the RNA polymerase II second largest subunit (RPB2), or translation elongation factor 1-alpha (TEF1-a) [20,34].

*Cytospora* species are primarily wound pathogens and cause cankers and dieback on many genera of hardwoods and coniferous trees, but rarely on herbaceous plants [18]. On many tree species, these fungi are considered facultative wound parasites that attack weakened trees [18]. Petrini [35] describes *Cytospora* spp. as latent pathogens existing as symptomless endophytic infections. The pathogens infect the inner bark, which is also referred to as the bark periderm [18]. *Cytospora pruinosa* Défago may infect olive trees through pruning wounds and wounds caused by cold. Abiotic stress may also play a role in decline [36]. The climatological conditions and the availability of susceptible hosts may influence the distribution of *Cytospora* species [5]. Plant pathologists are concerned that pathogens such as species of *Cytospora* might move from an introduced host to a native host or vice versa [18]. Because *Cytospora* species cause disease in plants under stress, the movement of a pathogen to a new host may reflect the vulnerability of a host’s defensive systems under stress or the severity of the stress [18]. Fruiting bodies of *Cytospora* spp. consist of stromata (conidiomata) that usually contain either labyrinthine chambers or clusters of pycnidia, having filamentous conidiophores and allantoid hyaline conidia [18]. Conidia oozing from pycnidia embedded in dead or dying host cortical tissues during humid or wet conditions are considered infectious propagules potentially initiating new infections. Conidia exude from the fruiting bodies in gelatinous matrices, usually as yellow, orange, red, or pallid tendrils [18]. They are dispersed to new plant tissues by rain-splash, where they germinate and infect the host through cracks and wounds to the bark created by breakage of shade-weakened twigs and branches, insect injuries, leaf scars, pruning wounds, winter-injured buds, twigs, and bark [31,37,38]. As plant pathogens, *Cytospora* species are primarily associated with canker diseases. Symptoms vary with host species and stage of development [26]. *Cytospora* species mainly impact branches, but they can cause more destructive infections in the trunk and larger scaffolds, consequently limiting the productivity of orchards [38,39]. The diseased inner bark and the bark above the infected cambium may appear sunken and yellow, brown, reddish-brown, grey, or black, becoming watery and odorous as the tissues deteriorate. The wood below the cambium is stained brown. Later, these fungi quickly girdle and kill branches and twigs, forming several black sporocarps [26].

The objective of this study was to identify the causal agent of canker disease on olive trees observed in two orchards in Istria, Croatia, based on morphological examination, phylogenetic analysis, and to confirm a pathogen is the cause of a particular disease using Koch’s postulates. 

## 2. Materials and Methods

### 2.1. Sampling and Fungal Isolation

In 2021, olive trees showing signs of dieback were observed in two olive orchards in Istria, Croatia. The first orchard was on the northern side of Istria, in Kaštelir (45°17′30″ N, 13°40′51″ E). The area of the orchard was 6.5 ha, with 44 different olive cultivars. Symptoms were observed on olive trees from the local cultivar ‘Porečka rosulja’. The second orchard was on the western side, in Vodnjan (44°56′21″ N, 13°50′18″ E). The area of this orchard was 1 ha and it contained two different olive cultivars. Symptoms were observed on olive trees of the local cultivar ‘Buža’. In both orchards, olive trees were between five and 20 years old. In total, five samples (one sample per tree) of branches from symptomatic trees were collected from each orchard and delivered to the Laboratory for Plant Protection at the Institute of Agriculture and Tourism in Poreč (Istria, Croatia) for analysis.

Whole olive fruits and small pieces of branches and leaves (5 × 5 mm) were rinsed under tap water, surface sterilized in 70% ethanol for one minute (two minutes for fruits), rinsed two times in sterile distilled water, and placed on a sterile paper sheet in a laminar flow cabinet until dry. Plant parts were plated on potato-dextrose agar (PDA) and incubated for seven days at 25 °C under laboratory conditions. After incubation, the growing tips of hyphae were transferred aseptically on the fresh PDA medium, for pure culture.

### 2.2. Morphological Identification

Pure fungal cultures of ten isolates on PDA were taken for examination. Species have been identified based on the spores (color, shape, presence or absence of septa, and dimensions) and colony characteristics (color, form, elevation, margin, surface, and opacity). A study of the fungal structure was performed with a Boeco BM-2000 microscope, a Boeco BCAM10 camera, and a B-View software (Boeckel + Co (GmbH + Co) KG, Hamburg, Germany). Morphometric values were compared with previously published data for the genus [26]. 

### 2.3. Molecular Identification and Phylogenetic Analyses

Two representative isolates, SL2 PRIV and V16 BIII (one per orchard), were chosen for molecular identification. To fully characterize the isolates, DNA sequences of internal transcribed spacer (ITS) and beta-tubulin (TUB) were determined. Fresh mycelia of fungal isolates grown on PDA for seven days at 25 °C were scraped with a sterile toothpick from the colony margins and used for genomic DNA extraction. Total genomic DNA from the isolate was extracted using a Maxwell^®^ RSC Instrument (Promega, Madison, WI, USA) and Maxwell^®^ RSC Plant DNA Kit (Promega, Madison, WI, USA). The nuclear ribosomal DNA repeats were amplified using ITS1 (5′ TCCGTAGGTGAACCTGCGG 3′) and ITS4 (5′ TCCTCCGCTTATTGATATGC 3′) pair of primers [40]. Oligonucleotide primers Bt2a (5′ GGTAACCAAATCGGTGCTGCTTTC 3′) and Bt2b (5′ ACCCTCAGTGTAGTGACCCTTGGC 3′) were used to amplify a portion of the TUB gene [41]. The polymerase chain reaction (PCR) mixture was composed of 12.5 µL of EmeraldAmp^®^ GT PCR Master Mix, 0.5 µL of each primer, 6.5 µL of nuclease-free water, and 5 µL of genomic DNA. PCR was conducted in a SureCycler 8800 Thermal Cycler (Agilent Technologies, Santa Clara, CA, USA) under the following conditions for both gene regions: initial denaturation step for two minutes at 95 °C followed by 35 cycles for 30 s of denaturation at 95 °C, 30 s for annealing at 48 °C, one minute for extension at 72 °C, and a final extension step of eight minutes at 72 °C [26]. For both isolates, the amplification of the TUB region was not accomplished, so a second PCR was performed using 1 µL of the first PCR amplification as a template. PCR products were visualized on 1% agarose gel light using an iBright CL1000 Imaging System (Invitrogen, Thermo Fisher Scientific, Waltham, MA, USA). Purification and sequencing of PCR products was conducted by Macrogen Europe services (Amsterdam, The Netherlands). Nucleotide sequences were read and edited in Sequencher^®^ (Gene Codes Corporation, Ann Arbor, MI, USA). Sequences were compared with those of *Cytospora* species from previous studies available in the National Center for Biotechnology Information database GenBank^®^. Consensus sequences were produced and deposited into GenBank^®^. Phylogenetic analysis was made using ITS sequence data from isolates used in this study and relevant isolates from GenBank^®^. Sequences were aligned using ClustalX2 (UCD Dublin, Dublin, Ireland) software, and a phylogenetic tree was made using MEGA11 (Pennsylvania State University, Center, PA, USA) software.

### 2.4. Pathogenicity Tests

To determine the pathogenicity of fungal species, the same two isolates selected for molecular analysis were chosen for the inoculation of olive branches. Two pathogenicity tests were performed: inoculation on detached branches and inoculation on olive trees.

#### 2.4.1. Pathogenicity on Detached Branches

In total, ten segments of branches per cultivar, 10 cm long, were collected from healthy olive trees of cultivars ‘Buža’, ‘Leccino’, and ‘Porečka rosulja’ grown at the Institute of Agriculture and Tourism in Poreč. Branch segments were rinsed under tap water, surface sterilized in 10% sodium hypochlorite for 10 min, and rinsed with sterile distilled water for 10 min. Segments were placed in a laminar flow cabinet on a sterile paper tissue. After air drying, the branch segments were marked and sealed at both ends with Parafilm to reduce desiccation. Wounds 4 mm in diameter were made in the bark with a cork borer to remove the outer bark but to leave the inner bark intact. A 4 mm diameter mycelium plug from an 8-day-old PDA culture of isolates was placed in each wound. Inoculated wounds were sealed with Vaseline and protected with Parafilm. Two replicates were performed. PDA plugs without mycelium were used as a control. Inoculated branches were placed in a plastic bag on a sterile paper tissue soaked with sterile distilled water, and incubated under laboratory conditions at approximately 21 °C for 10 weeks.

#### 2.4.2. Pathogenicity on Olive Trees

Branches of 2-year-old olive trees of cultivars ‘Buža’, ‘Leccino’, and ‘Porečka rosulja’ were inoculated in a greenhouse at the Institute of Agriculture and Tourism in Poreč. Four branches per replicate, per cultivar were inoculated for each isolate. Branches were disinfected with 70% ethanol, and 4 mm diameter wounds were made in the bark with a cork borer and inoculated the same way as previously described for detached branches. Inoculated plants had been kept in a greenhouse, at approximately 25 °C, for 6 months, from March to September 2022, and monitored for the presence of symptoms. After incubation, samples were collected. In an attempt to fulfill Koch’s postulate, small pieces of necrotic tissue from the edge of the developed lesion were placed on a PDA medium to reisolate the inoculated fungus. 

## 3. Results

### 3.1. Sampling and Fungal Isolation

**Figure 1:**
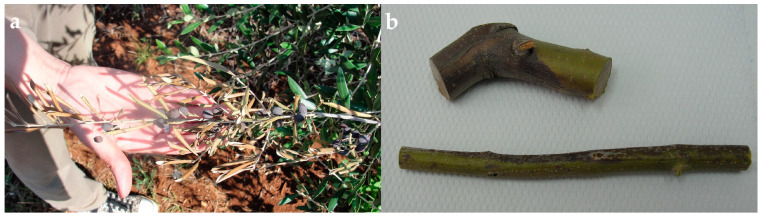
(**a**) Disease symptoms on olive tree in orchard. (**b**) Branch segment with bark discoloration taken for analysis.

The symptoms of the disease in the field can be described as the dieback of twigs and branches (Figure 1), brown internal necrosis on branches and under the bark, leaf necrosis, and fruit dieback. The symptoms were observed on trees from cultivars ‘Porečka rosulja’ and ‘Buža’. Symptoms affect only part of the trees. Morphologically similar fungal colonies were retrieved from all 10 samples. 

### 3.2. Morphological Identification

**Figure 2:**
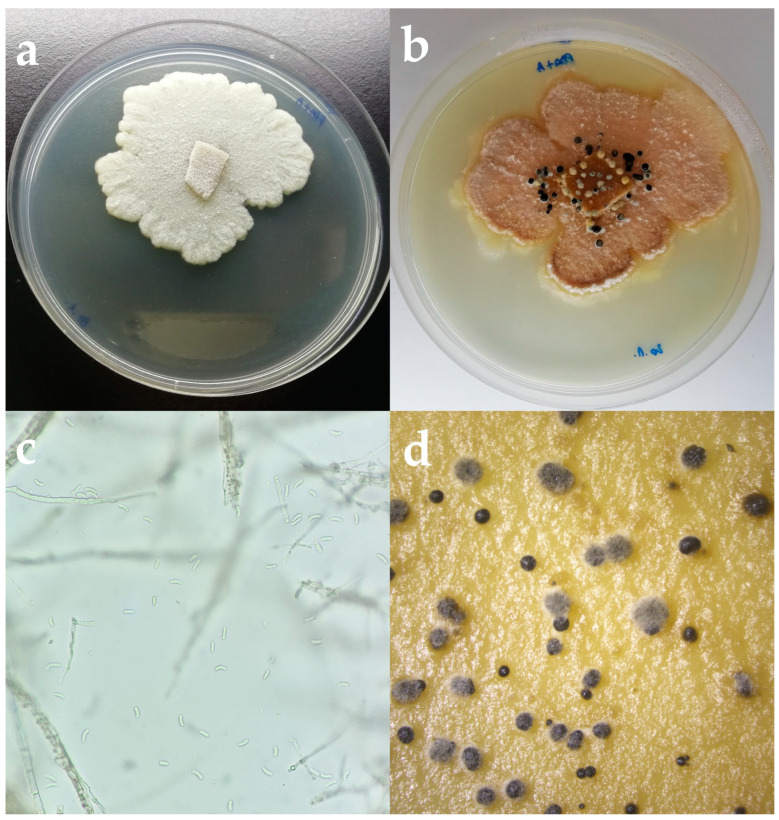
(**a**) *Cytospora pruinosa* colony on PDA after 5 days in the dark at 25 °C. (**b**) *C. pruinosa* colony on PDA after 2 weeks. (**c**) Hyphae and spores of *C. pruinosa* isolate under the microscope. Scale bar = 10 µm. (**d**) Conidiomata formed on PDA.

Based on the colony and spore characteristics, 10 fungal isolates have been identified as *Cytospora pruinosa* Défago. Colonies developing on PDA had reached a nine cm diameter after 10 days at 25 °C on PDA. Colonies were irregularly shaped, with aerial, opaque, fluffy mycelium becoming flat with age. Initially, they were white becoming creamy white-beige with age with black pycnidia distributed on the surface (Figure 2). The reverse of the colonies was creamy white with an orange undertone and visible black pycnidia. Hyphae were septate, hyaline, and yellowish. Conidia were elongated, hyaline, aseptate, and smooth, and 5.6–6.8 × 1.1–1.3 µm diameter ($\bar{x}$ = 5.9 × 1.1 µm, *n* = 30).

### 3.3. Molecular Identification and Phylogenetic Analyses

**Figure 3:**
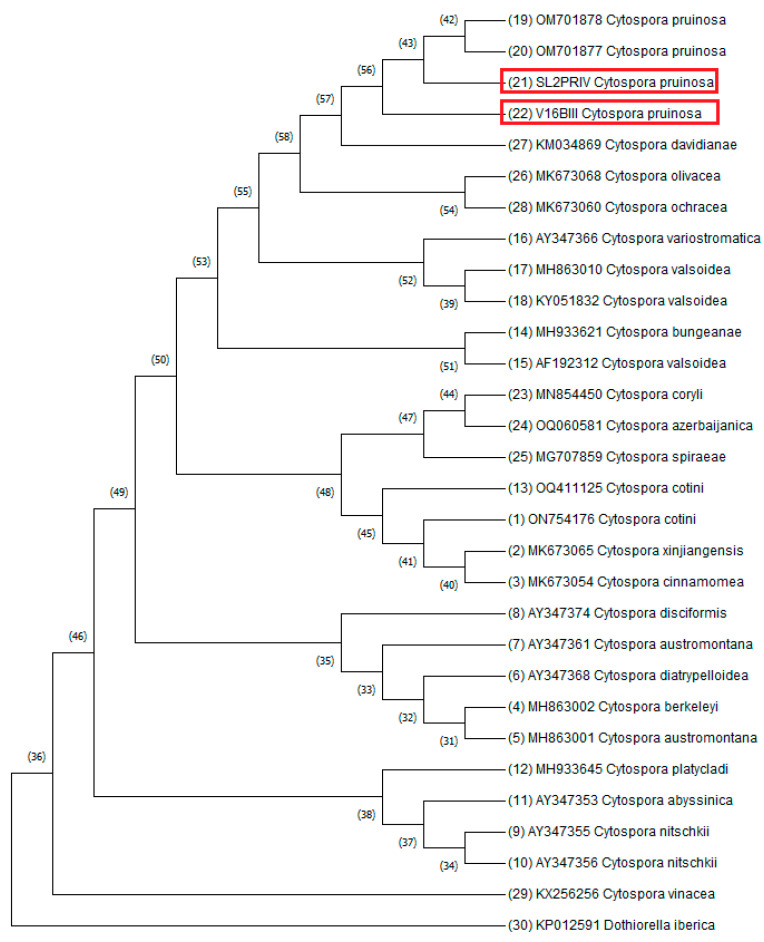
The evolutionary history was inferred using the Neighbor-Joining method [42]. The optimal tree is shown. The percentage of replicate trees in which the associated taxa clustered together in the bootstrap test (1000 replicates) is shown next to the branches [43]. The evolutionary distances were computed using the Maximum Composite Likelihood method [44] and are in the units of the number of base substitutions per site. This analysis involved 30 nucleotide sequences. *Dothiorella iberica* isolate 211 KP012591 was used as an outgroup. Sequences from this research are marked with red rectangles. All ambiguous positions were removed for each sequence pair (pairwise deletion option). There were a total of 769 positions in the final dataset. Evolutionary analyses were conducted in MEGA11 [45].

Consensus sequences of representative isolates were produced and deposited in GenBank^®^ under accession numbers: OQ642321 and OQ644501 for ITS, and OQ652101 and OQ694815 for the TUB gene region (available in Supplementary Materials). Blast analysis of the sequences from the SL2 PRIV isolate showed 100% similarity for ITS and TUB gene regions with *C. pruinosa*. Blast analysis of the sequence from the V16 BIII isolate showed 100% similarity for ITS and 99.79% similarity for the TUB gene region with *C. pruinosa*. The phylogenetic tree was constructed using the Neighbor-Joining method (Figure 3). DNA sequence analysis and phylogenetic analysis confirm the identity of SL2 PRI and V16 BIII isolates as *C. pruinosa.*

### 3.4. Pathogenicity Tests of Isolate

**Figure 4:**
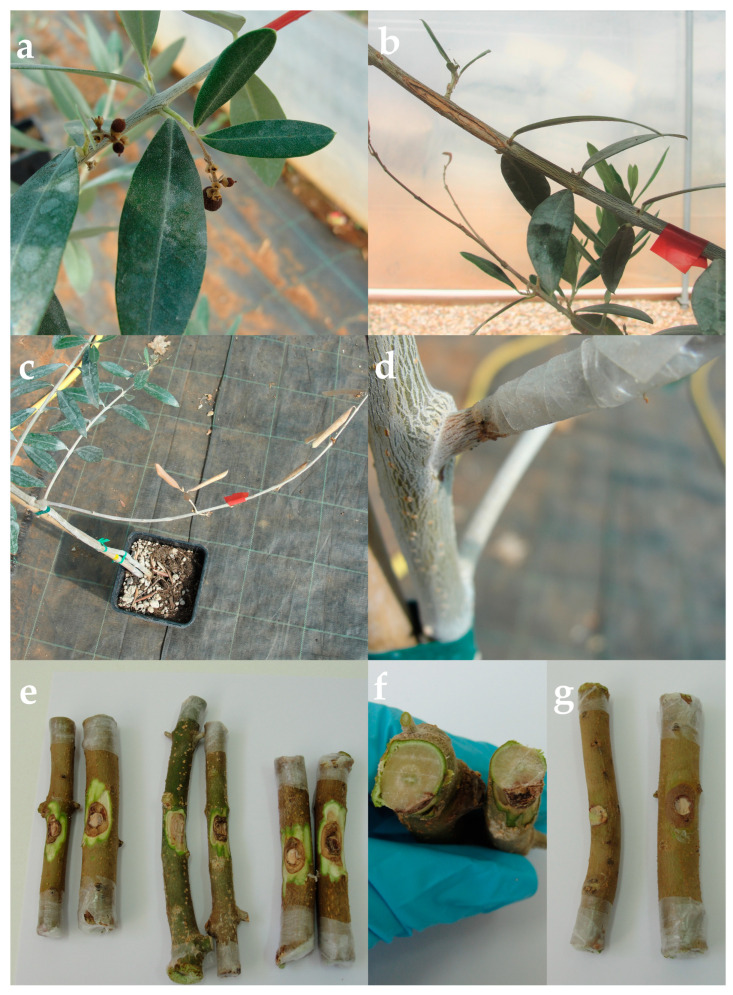
Disease symptoms on olive trees used in pathogenicity tests in the greenhouse after 6 months at 25 °C: (**a**) fruit collapse, (**b**) branch necrosis, (**c**) branch dieback, (**d**) bark discoloration. (**e**,**f**) Disease symptoms on olive branches used in pathogenicity tests in the laboratory: (**g**) Difference between the control branch inoculated with pure PDA plug (left) and the branch inoculated with *C. pruinosa* (right).

The symptoms of the disease on the olive branches tested in the laboratory and on the olive trees in the greenhouse showed the same symptoms as olive trees observed in the field survey. Dieback of twigs, brown internal necrosis on branches and under the bark, bark discoloration, and fruit collapse were detected (Figure 4). The pathogen had been consistently reisolated from all affected pieces of wood. Only saprobes were isolated from the control branches and trees.

## 4. Discussion

In this research, *C. pruinosa* was identified based on morphological characteristics, molecular data of ITS and TUB gene region, and a phylogenetic tree made based on internal transcribed spacer sequence alignment. Pathogenicity tests were conducted on detached olive branches and whole plants from three different olive cultivars. Cultivars ‘Buža’ and ‘Porečka rosulja’ were chosen for the test because all isolates were derived from those olive cultivars. Cultivar ‘Leccino’ was chosen as one of the most distributed and resistant olive cultivars in Istria. Lesions appeared on all tested branches and plants, except for the controls. In addition, Koch’s postulate was carried out on all infected and control plants. Disease symptoms of infection of olive trees inoculated with *C. pruinosa* comprised reddish-brown discoloration of bark, stained brown discoloration below the cambium, leaf necrosis, branch, fruit, and twig dieback.

Members of *Cytospora* genus are cosmopolitan and occur on a broad host range [26]. The first record of *C. pruinosa* on olives was in 2006 in South Africa [18]. In addition to Africa [18,46] it was found as a pathogen of olives in Spain [11]. Other *Cytospora* species known as olive pathogens are *C. oleicola* D.P. Lawr., L.A. Holland & Trouillas [5,31], *C. oleina* Berl. [47], *C. olivarum* Úrbez-Torr., D.P. Lawr., Peduto, Gubler & Trouillas [5], *C. plurivora* D.P. Lawr., L.A. Holland & Trouillas, and *C. sorbicola* Norphanph., Bulgakov, T.C. Wen & K.D. Hyde [31]. Except dieback of olives, *C. pruinosa* is associated with dieback of ash tree *Fraxinus excelsior* L. *C. pruinosa* can infect ash trees weakened after primary infection by fungus *Hymenoscyphis fraxineus* (T. Kowalski) Baral, Queloz & Hosoya, or emerald ash borer *Agrilus planipennis* Fairmaire [48,49].

Moral et al. [11] reinforce the idea that inoculation in vivo is essential for the characterization of fungal pathogens. In their trial, *C. pruinosa* did not cause symptoms when tested on 5-year-old potted ‘Gordal Sevilliana’ olive trees in a greenhouse at 25 to 30 °C. Contradictory to their study, *C. pruinosa* formed the largest average lesion length of all the isolates used in a study conducted in 2021 in South Africa [46]. However, of the six tested isolates of *C. pruinosa*, two isolates developed long lesions with values of 41.14 mm and 36.94 mm, one isolate with 10.53 mm, while the rest had values of less than 10 mm. In total, of 58 fungal isolates comprising 38 species, *C. pruinosa* produced not only the longest lesions but also one isolate was on the bottom 13 isolates with an average lesion length of only 3.20 mm [46]. In the research carried out by Úrbez-Torres et al. [5], six months after inoculation of olive branches, *C. oleicola* and *C. olivarum* caused lesions that averaged 26.7 mm in length. All tested isolates of *C. oleina* in spring inoculations resulted in the formation of a necrotic area around the inoculation point 43 to 56 mm long, while inoculation made in autumn resulted in the death of the whole twig [47]. In this trial, six months after inoculation of trees, *C. pruinosa* caused lesions that averaged 10.4 mm on cv. Porečka rosulja, 22.4 mm on cv. Leccino, and 31.36 mm on cv. Buža. Compared to other fungal pathogens, such as species from the *Botryosphaeriaceae* family, *Cytospora* species were shown as less aggressive pathogens.

Inability to form symptoms could be explained by *C. pruinosa* previously being part of a species complex with genetic variances expected to be high. These variances could create implications regarding the variation in virulence-related genes [18,46].

Control of *Cytospora* diseases is difficult and focusing management efforts against the most aggressive encountered *Cytospora* species will be essential [31]. Preventive practices, such as proper pruning and pruning of infected parts, clean tools, removal of infected plant material from orchards, treatment of pruning wounds, and selection of resistant cultivars may be an effective preventative strategy against infection with *Cytospora* species. Mechanical pruning results in multiple cuts on trees, which greatly surpass the number of pruning wounds produced in traditionally farmed low-density orchards, so further research needs to be carried out to investigate the impact of mechanized practices on infection by *Cytospora* spp. [5].

There is no data about protection measures for *C. pruinosa* exclusively; protection measures against other *Cytospora* species have been listed. Regarding fungicides, thiophanate-methyl (alone, amended in 50% latex paint, combined with VitiSeal, and combined with latex paint at 50 and 70%), captan, 50% latex paint, lime sulfur, and VitiSeal combined with lime sulfur has the potential for reducing the species *C. leucostoma*, *C. plurivora*, and *C. sorbicola* [50,51,52]. In addition, antagonistic fungal species *Trichoderma viride* SC1 provide excellent pruning wound protection against *C. sorbicola* [51]. Moreover, antagonistic assays in vitro showed that secondary metabolites of *Bacillus pumilus* Meyer and Gottheil strain JK-SX001 extracted using methylbenzene could also suppress the growth of *C. chrysosperma* (Pers.) Fr. [53].

Since there is a lack of information about the biology and pathogenicity of this species, further research needs to be carried out. Urbez-Torres [5] propose research based on spore-trapping studies combined with studies evaluating the effectiveness of pruning wound protectants for development of effective control strategies. *Cytospora* species has not been recorded on other plant species in Croatia so far. Given that spores of some of the *Cytospora* species, such as *C. leucostoma*, can be wind-blown to 76 m from the inoculum source [54], and given that *Cytospora* species are pathogens of numerous plant species, especially woods, the question arises as to the possibilities of their spread on other plants, mostly on vines, because vines and olives are often grown together in these areas.

## 5. Conclusions

To the best of our knowledge, this is the first report of *Cytospora pruinosa* Défago causing olive twig and branch dieback on olive trees in Croatia.

## Data Availability

All sequence data are available in NCBI GenBank following the accession numbers in the manuscript.

## Supplementary Materials

The following supporting information can be downloaded at: https://www.mdpi.com/article/10.3390/microorganisms11071679/s1. Flatfile records of the isolates from NCBI.
